# Post-masking in cortical auditory evoked potentials following acoustically controlled auditory training: a case report

**DOI:** 10.1590/2317-1782/e20250206en

**Published:** 2026-04-10

**Authors:** Matheus Costa Gonçalves, Pedro de Lemos Menezes, Carlos Henrique Alves Batista, Danielle Cavalcante Ferreira, Kelly Cristina Lira de Andrade

**Affiliations:** 1 Universidade Estadual de Ciências da Saúde de Alagoas – UNCISAL – Maceió (AL), Brasil.

**Keywords:** Auditory Perception, Auditory Perceptual Disorders, Auditory Evoked Potential, Noise, Speech

## Abstract

Central auditory processing refers to the individual's ability to process auditory information within the central auditory nervous system and involves a set of auditory skills. Deficits in one or more of these skills characterize central auditory processing disorder. Given the presence of this disorder, auditory training is indicated as a set of strategies designed to activate the auditory system and improve the processing of acoustic signals. Electrophysiological measures such as cortical auditory evoked potential may serve as a valuable supplement to the behavioral assessment of auditory processing. When associated with noise, temporal masking is assessed. It is characterized as an alteration in the perception of a sound in the presence of another auditory stimulus. Individuals with this disorder are more susceptible to post-masking effects due to difficulties in understanding speech in noisy environments. This study reports the case of a 25-year-old adult diagnosed with central auditory processing disorder who underwent cortical auditory evoked potentials using speech stimulus preceded by noise. The acoustically controlled auditory training was conducted over 10 sessions, using the “Afinando o Cérebro” platform. Following intervention, improvements were observed in the participant's auditory skills, along with reductions in the latencies of the P1, N1 and P2 waves and in the N1-P1 and N1-P2 peak-to-peak amplitudes. These findings highlight the effects of acoustically controlled auditory training and contribute to a better understanding of the neurophysiological mechanisms involved in speech perception in noise among individuals with central auditory processing disorder.

## INTRODUCTION

Central Auditory Processing (CAP) refers to the individual's ability to process auditory information in the Central Auditory Nervous System (CANS) and is composed of a set of auditory skills, including sound localization and lateralization; auditory discrimination; auditory pattern recognition; temporal aspects of hearing (temporal resolution, temporal masking, temporal integration and temporal ordering); auditory figure-ground differentiation and; auditory closure. Deficits in one or more of these skills characterize Central Auditory Processing Disorder (CAPD)^(1)^.

CAPD is a neurobiological disorder that affects auditory perception and may coexist with other disorders. However, it should not be considered a secondary manifestation of global deficits. Therefore, accurate diagnosis requires differential evaluation, and the assessment of CAP should be conducted through behavioral and electrophysiological tests^(2)^.

In the context of CAPD, auditory training is indicated and is defined as a set of strategies that activate the auditory system, promoting changes in the neural substrate and auditory behavior, thereby improving the processing of acoustic signals^(3)^. Acoustically Controlled Auditory Training (ACAT) refers to the implementation of these strategies in acoustically favorable environments where stimuli are delivered in a controlled manner using audiometers and computer-based systems^(2,3)^.

Electrophysiological measures such as the Cortical Auditory Evoked Potential (CAEP) can be used to complement CAP assessment. CAEP represent long-latency neuroelectric responses that reflect thalamo-cortical activity and are composed of the P1-N1-P2 waveform complex, which marks the arrival of auditory information at the auditory cortex^(4)^.

When combined with noise, this potential allows for the assessment of temporal masking, which is characterized by an alteration in the perception of one sound in the presence of another auditory stimulus^(5)^. This noise can occur in three forms: (1) simultaneously with the target sound, resulting in simultaneous masking*;* (2) after the target sound, resulting in backward masking; and (3) before the target sound, resulting in post-masking or forward masking^(6)^.

Post-masking, the focus of this study, is defined as the persistence of the masking effect of noise for a few milliseconds (ms) after its onset. This effect can interfere with the perception of subsequent stimuli, particularly speech^(7)^. The time interval between the noise and the speech stimulus influences the post-masking effect, presenting an inversely proportional relationship, i.e., the shorter the interval between the stimuli, the greater the post-masking effect^(6)^.

Individuals with CAPD are believed to be more susceptible to post-masking effects due to their difficulties in understanding speech in the presence of noise^(4)^. However, ACAT has the potential to reduce these effects, and its effectiveness can be assessed using CAEP elicited by noise and speech stimulus. As CAEP are exogenous potentials, which are independent of the participant's responses, they provide objective insights into speech perception in noise.

In this scenario, the objective of this study was to report the case of a 25-year-old adult diagnosed with CAPD who underwent ACAT and was evaluated from CAEP with masking and without masking, using speech stimulus followed by at different signal-to-noise ratios, before and after intervention.

## CLINICAL CASE PRESENTATION

This is a case report developed in a research laboratory linked to a public university located in the city of Maceió/AL, approved by the institution's research ethics committee.

A 25-year-old male adult who complained of hearing difficulties in noisy environments, especially during group conversations, was invited to participate in the study. The participant reported being inattentive in addition to having had difficulties in school during childhood, especially in the early grades. The participant is currently a medical student. He has no health complaints.

The selection of this participant for the present case report is based on the clinical relevance of their symptomatology and on clear evidence of specific deficits in central auditory processing skills, particularly auditory closure and temporal processing. Deficits in these auditory functions are widely recognized as being associated with reduced speech intelligibility in acoustically adverse environments, significantly impacting functional communication. Thus, the participant’s audiological profile strategically aligns with the objectives of this investigation, enabling a detailed analysis of the correlations between the reported subjective perception and the potential underlying deficits in central auditory processing, thereby contributing to a better understanding of the involved pathophysiological mechanisms.

Initially, the objectives and collection procedures were explained, and the Free and Informed Consent Form (FICF) was subsequently signed. The procedures were divided into pre-collection and research procedures, described as follows:

### Pre-collection procedures

The participant initially underwent cognitive screening using the Montreal Cognitive Assessment (MoCA)*,* in which he scored 28 points. According to standard criteria, a score of 26 or higher is considered within the normal limits, with a maximum possible score of 30. Otoscope inspection of the ear canal and tympanic membrane was then performed, showing no abnormalities. A basic audiological evaluation was performed, including pure-tone audiometry, speech audiometry and immittance testing. Pure-tone and speech thresholds within normal standards were identified, a Type A tympanogram and ipsilateral and contralateral acoustic reflexes.

For the brainstem auditory evoked potentials (BAEP), skin preparation was performed using NUPREP® abrasive paste. Disk-type electrodes were placed with conductive paste according to the International 10–20 System at the following positions: active electrode at Cz (vertex), reference electrode at M2 (right mastoid), and ground electrode at Fpz (forehead). Stimuli were delivered to the right ear via ER-3A insert earphones. Electrode impedance values were kept at or below 3 kΩ, and artifact rejection remained under 10% of recorded sweeps.

The recording parameters for this potential were as follows: click stimulus; 10 ms recording window; stimulation rate of 21.1 clicks/second; band-pass filter of 100–3000 Hz; gain of 100,000; stimulus duration of 100 µsec; averaging of 1500 to 2000 stimuli; rarefaction polarity; and intensity of 80 dB nHL. Waves I, III, and V were identified, with absolute latencies and interpeak intervals I–III, III–V, and I–V within normative values, as well as an interaural wave V latency difference (V–V) of less than 0.3 ms.

### Collection procedures

After confirming results within normal limits in the previous assessments, CAP behavioral assessment was initiated using the following tests: Sound localization test; Sequential memory test for verbal and nonverbal sounds; Synthetic Sentence Identification Test with Ipsilateral Competing Message (SSI/ICM) at signal-to-noise ratios (SNR) of 0 and -15 dB; Dichotic Digit Test (DDT) in free attention mode; Pitch Pattern Sequence Test (PPS), developed by Auditec; Binaural Fusion Test; Random Gap Detection Test (RGDT).

Altered performance was observed in the following tests: SSI/ICM, DDT, PPS and RGDT. Poor performance on these tests reflects deficits in the auditory skills of figure-ground for verbal sounds associated with visual stimuli, binaural integration, temporal ordering, and temporal resolution, respectively.

The CAEP recording followed the same electrode placement and equipment used in the BAEP. The syllable /ba/ and a formatted speech-shaped noise (in the masking conditions) were presented monaurally to the right ear through insert earphones (ER-3A) across five randomized test conditions: without masking and with masking at the inter-stimulus interval (𝚫t) of 3 ms under SNR of 0, -10, -20 and -30 dB.

Electrophysiological assessments were conducted exclusively in the right ear, based on specific neuroanatomical and methodological considerations. Given that the research protocol employed speech stimuli as the primary target, the choice of right-ear stimulation is justified by the cerebral hemispheric organization, in which language processing is predominantly lateralized to the left hemisphere. Thus, the contralateral auditory pathway (right ear → left hemisphere) provides privileged access to neural circuits specialized in the processing of verbal stimuli, optimizing the detection of training-induced cortical changes.

The syllable /ba/ was adapted from a previous study^(8)^, but truncated to a duration of 85 ms, with the waveform ending in a zero-crossing point. The original sampling rate of 11,025 Hz was adjusted to the rate of 48,000 Hz, as required by the Biologic® Navigator PRO system used in this study. The stimulus level was calibrated in reference to the dB SPL of a 1 kHz continuous tone that had the same peak-to-peak amplitude as the /ba/ waveform; i.e., dB SPL. The masker consisted of a speech-shaped noise (SSN) with the same long-term average spectrum as multilingual speech. Its total duration was 300 ms, including a 10 ms onset ramp and a 40 ms offset ramp. These ramps durations were selected based on pilot testing, which indicated that this onset was abrupt enough to elicit a detectable CAEP recording, but not an offset, i.e., the masker elicited an onset-evoked CAEP, but not an offset-evoked response. The masker level was calibrated in dB SPL.

In the without-masking condition, the syllable /ba/ was presented at 70 dB nHL. In the masking conditions, the syllable /ba/ was presented at levels ranging from 70 and 40 dB nHL, according to the signal-to-noise ratio, while the noise was presented 3 ms prior to the syllable /ba/ fixed at 70 dB nHL. In all conditions, 140 stimuli were delivered at a rate of 0.7 stimuli per second, using alternating polarity, a band-pass filter of 1-30 Hz, and a recording window of 799.5 ms.

The participant was invited to watch a short, black-and-white, soundless film to maintain alertness during CAEP acquisition. Regarding the waveforms, the P1, N1 and P2 components were identified, and their latencies and peak-to-peak amplitudes were analyzed. To ensure the reliability of the results, two researchers experienced in the area independently analyzed the waveforms and marked the components. Both were blinded to the test condition. In cases of disagreement regarding the marking of any component, a third researcher was consulted for consensus.

The ACAT was performed in a soundproof booth and with supra-aural headphones. The participant underwent 10 sessions, twice a week, which lasted 40 to 60 minutes each. The digital platform “Afinando o Cérebro” by ProBrain®, developed to stimulate auditory, visual and cognitive skills, was used to structure the sessions. The monotic and dichotic listening activities were delivered, respectively, at 40 and 50 dB above the speech recognition threshold.

During the sessions, more than one auditory skill was stimulated, progressively increasing the level of difficulty. In tasks where performance was 70% or higher, the level of difficulty was increased. If performance was 30% or below, the level of difficulty should be reduced. The activities performed in the sessions are described in Chart 1.

**Chart 1:** Activities carried out during Acoustically Controlled Auditory Training sessions

**Sessions**	**Figure-ground**	**Binaural integration**	**Temporal resolution**	**Temporal ordering**
**1st**	Figure-background categorization	Integrating (level 1)	Heart monitor	Strong/weak
**2nd**	Figure-background categorization	Integrating (all levels)	Time perception (levels 1 and 2)	Ordering/frequency
**3rd**	Searching (all levels)	Integration question	Time perception (all levels)	Frequency interval 5th
**4th**	Multi audios (levels 1, 2 and 3)	Integration question	Count the sounds	Frequency interval 5th
**5th**	Multi audios (all levels)	Dichotic discrimination	Drops (level 1 and 2)	Frequency interval 5th
**6th**	Connection failed	Binaural Digit Integration (Levels 1 and 2)	Drops (all levels)	Frequency interval 3rd
**7th**	Connection failed	Binaural Digit Integration (all levels)	Mr. Stressed (Levels 1, 2 and 3)	Frequency interval 3rd
**8th**	Restaurant (level 1)	Word Challenge (Level 1)	Mr. Stressed (all levels)	Following the notes (level 1)
**9th**	Restaurant (levels 1 and 2)	Word Challenge (Level 1)	Hard Heart Monitor (level 1)	Following the notes (levels 1 and 2)
**10th**	Restaurant (all levels)	Word Challenge (Levels 1 and 2)	Hard Heart Monitor (all levels)	Following the notes (all levels)

**Caption:** ACAT: Acoustically controlled auditory training

Two weeks after completing ACAT, the participant underwent a reassessment of both CAP and CAEP following the specifications of the initial assessment. The results of the pre- and post-ACAT assessments, along with the normative values for each behavioral test are shown in Chart 2. Table 1 displays CAEP results before and after ACAT.

**Chart 2:** Results of Central Auditory Processing behavioral assessment before and after Acoustically Controlled Auditory Training

**Tests**	**Ear**	**Time point**	**Results**	**Normative Standard**
**Sound localization**	Binaural	Pre-therapy Post-therapy	5 correct	≥ 4 correct
			5 correct	
**Nonverbal sequential memory**	Binaural	Pre-therapy Post-therapy	3 correct	≥ 2 correct
			3 correct	
**Verbal sequential memory**	Binaural	Pre-therapy Post-therapy	3 correct	≥ 2 correct
			3 correct	
**SSI/ICM**	RE SNR 0 dB	Pre-therapy Post-therapy	70%	SNR 0 dB = 80%
			80%	
	LE SNR 0 dB	Pre-therapy Post-therapy	70%	
			80%	
	RE SNR -15 dB	Pre-therapy Post-therapy	50%	SNR -15 dB = 60%
			70%	
	LE SNR -15 dB	Pre-therapy Post-therapy	50%	
			80%	
**Binaural fusion**	RE	Pre-therapy Post-therapy	92%	≥ 80% in both ears
			92%	
	LE	Pre-therapy Post-therapy	96%	
			96%	
**RGDT**	Binaural	Pre-therapy Post-therapy	12.5 ms	≤ 10 ms
			8.75 ms	
**DDT (directed attention)**	RE	Pre-therapy Post-therapy	87.5%	≥ 95% in both ears(≥11 years)
			97.5%	
	LE	Pre-therapy Post-therapy	87%	
			95%	
**PPS**	Binaural	Pre-therapy Post-therapy	56.66%	≥ 75% (≥ 11 years)
			76.66%	

**Caption:** % = Percentage; RE = Right ear; LE = Left ear; dB = Decibels; SSI/ICM = Synthetic sentence identification with ipsilateral competing message; RGDT = Random Gap Detection Test; DDT = Dichotic digit test; PPS = Pitch Pattern Sequence

**Table 1:** Results of peak-to-peak latencies and amplitudes of P1, N1 and P2 waves, pre and post Acoustically Controlled Auditory Training

**Conditions**	**Signal to noise ratio**	**Moments**	**Latencies (ms)**			**Amplitude (µV)**	
			**P1**	**N1**	**P2**	**P1-N1**	**N1-P2**
Non-masking	Not applicable	Pre-therapy	347.01	406.63	485.74	6.07	6.60
		Post-therapy	333.04	397.20	452.19	3.97	5.61
Masking	0 dB	Pre-therapy	362.96	415.70	494.81	4.05	8.16
		Post-therapy	355.95	405.22	487.71	2.28	7.91
	-10 dB	Pre-therapy	373.38	421.53	499.49	4.12	7.43
		Post-therapy	371.99	413.24	472.82	2.36	5.83
	-20 dB	Pre-therapy	389.43	428.41	507.52	1.95	3.43
		Post-therapy	368.55	413.24	469.38	2.40	5.37
	-30 dB	Pre-therapy	388.29	430.71	508.67	2.16	5.04
		Post-therapy	385.74	429.28	502.61	1.51	2.76

**Caption:** dB = Decibels; ms = milliseconds; µV = microvolts

## DISCUSSION

In this study, improved performance was observed in the participant's auditory skills following ACAT, achieving results within normal limits across all tests used in the behavioral assessment. A previous study that also employed ACAT with a similar number, frequency, and duration of sessions reported significant improvements in auditory skills among adults and children with CAPD^(9)^. These findings suggest that AT can stimulate neural structures associated with the performance of trained auditory skills^(10)^.

The use of software in auditory training enables individualized recording and management of therapeutic outcomes. In this study, the “Afinando o cérebro” platform was well accepted by the participant and proved effective in stimulating altered auditory skills. A study conducted with children with CAPD associated with reading and writing difficulties also found this to be effective in stimulating these skills^(11)^. These findings suggest that the platform is an effective instrument for working on auditory skills across different age groups.

It is recommended that the behavioral assessment of CAP be complemented by electrophysiological tests, such as CAEP^(4)^. CAEPs provide information on the input and processing of sound stimuli at the auditory cortex level, and when noise is paired with the target stimulus, it is possible to evaluate the post-masking effect^(5)^.

Post-masking can be explained by neural adaptation, which is characterized by a reduction in neural response following the presentation of sound stimuli, reflecting decreased recruitment of neurons capable of responding to a subsequent stimulus^(12)^. Another possible explanation is that the cochlea’s temporary inability to recover its sensitivity after recent stimulation, resulting in a cumulative response within this structure^(5)^. Thus, post-masking yields data on temporal auditory processing and the auditory skill of figure-ground perception, contributing to the understanding of speech perception in noise^(7)^.

The most commonly used parameters for analyzing CAEP recordings are latency and amplitude. Latency corresponds to the time between the presentation of the sound stimulus and the appearance of the wave, measured in ms^(4)^. Amplitude, in turn, reflects the magnitude of synchronous neural activity, measured in microvolts, and can be influenced by the number of neurons^(13 ).^

In the present study, a decrease in the latencies of the P1-N1-P2 complex was observed after ACAT in all CAEP test conditions. A study^(11)^ conducted with 54 children with reading and writing difficulties used tone burst and speech stimuli to compare auditory performance pre and post intervention, and found a decrease in the latencies of the P1-N1-P2-N2-P300 complex following ACAT. Despite the similar results, it is important to highlight that the protocol used by the author of the referenced study differs from that employed in this study, especially regarding the use of noise preceding the speech stimulus.

The P1, N1 and P2 components of the CAEP are responsible, respectively, for the processes of encoding, decoding and discriminating the acoustic characteristics of sounds. Thus, reductions in latencies suggest better precision of the CANS, reflecting shorter processing time and improved speech perception^(14)^.

For this study, peak-to-peak amplitude was used, characterized by the voltage between the N1 component and any surrounding positive peak^(15)^. A reduction in all amplitudes was observed following ACAT, except in the -20 dB SNR (P1-N1 and N1-P2). The decrease in amplitudes following ACAT can be explained by a reduced recruitment of neurons and, consequently, less effort to encode, decode and discriminate the acoustic characteristics of sounds, even in the presence of noise preceding the target stimulus^(13)^.

In this study, a decrease in the post-masking effect was observed due to a reduction in CAEP latencies and amplitudes following ACAT. This indicated an improvement in temporal masking and, consequently, better speech perception in noise. Furthermore, the changes observed in CAEP reflected the improvement in behavioral tests.

The application of the post-masking protocol before and after ACAT provides electrophysiological markers that are sensitive to training-induced changes, overcoming limitations of traditional behavioral methods, which may be influenced by subjective factors and learning effects. The ability to objectively quantify improvements in auditory skills through cortical responses to the noise-speech paradigm represents a promising diagnostic tool for validating therapeutic interventions, establishing specific neural biomarkers for monitoring the effectiveness of auditory training. This methodological innovation holds the potential to transform clinical audiology practice by providing robust neurophysiological evidence of the benefits of ACAT and guiding evidence-based auditory rehabilitation protocols.

One limitation of this study concerns the absence of self-perception questionnaires related to CAP at the pre- and post-ACAT time points. These questionnaires are important tools for comparing the participant's perceived auditory performance in daily life before and after the intervention.

The unprecedented nature of this study presents new possibilities for understanding the effectiveness of ACAT and the effects of post-masking, offering valuable insights for future research. The findings may serve as a basis for broader investigations, especially randomized clinical trials, which could deepen and validate the presented findings, expanding the prospects for application in clinical practice.

## FINAL COMMENTS

The effectiveness of ACAT was demonstrated through the comparison of behavioral CAP results before and after therapy, as well as by the reduction in latencies and peak-to-peak amplitudes of the P1, N1 and P2 components of the CAEP with and without masking conditions, in which the noise preceding the speech stimulus was used at different signal-to-noise ratios.
